# The effect of exogenous melatonin and melatonin receptor agonists on intensive care unit and hospital length of stay: A systematic review and meta-analysis

**DOI:** 10.1371/journal.pone.0332031

**Published:** 2025-09-08

**Authors:** Art Burgess Kelleher, Mark O’Donovan, Deborah O’Doherty, Ros Lavery, Elaine Lehane, Mohamad M. Saab

**Affiliations:** 1 Catherine McAuley School of Nursing and Midwifery, University College Cork, Cork, Ireland; 2 College of Medicine and Health, University College Cork, Cork, Ireland; 3 Oncology Department, Beaumont Hospital, Dublin, Ireland; 4 Mater Private Hospital, City Gate, Mahon, Cork, Ireland; Southern Medical University Nanfang Hospital, CHINA

## Abstract

**Introduction:**

Melatonin supplements and melatonin receptor agonists are linked to reduced delirium in the Intensive Care Unit (ICU) which we hypothesised may affect the length of stay (LOS) in ICU or in hospital. In this review, we identified and critically appraised the literature on the effect of exogenous melatonin and melatonin receptor agonists on the ICU and/or hospital LOS among adults admitted to the ICU.

**Methods:**

Six electronic databases and three trial registries were searched for randomised controlled trials (RCTs). Screening, risk of bias assessment, quality appraisal, and level of evidence assessment were conducted and cross-checked by two reviewers independently. Meta-analyses with disease-specific subgroups were conducted to assess the mean difference in LOS for exogenous melatonin and melatonin receptor agonists compared with a placebo.

**Results:**

Twenty RCTs were reviewed with 14 having a low risk of bias. For ICU LOS (18 studies) there was significant statistical heterogeneity (I^2^ = 73%); compared with placebo the 95% prediction interval for the mean difference was −3.18 and 1.39 days. For hospital stay (12 studies, I^2^ = 79%) the 95% prediction interval ranged from −6.68 to 3.52. Removing two statistical outliers, and correcting for publication bias, there was no overall statistically significant difference in mean ICU LOS (*p*-value = 0.298) or mean hospital LOS (*p*-value = 0.456). The subgroup analysis found statistically significant improvements for those who underwent coronary artery bypass graft surgery (ICU LOS −0.47 days, 95% CI: −0.78 to −0.16, *p*-value = 0.003); and patients with COVID-19 (hospital LOS −3.90 days, 95% CI: −6.28 to −1.51, *p*-value = 0.001).

**Conclusion:**

There was a very low certainty of evidence that melatonin and melatonin receptor agonists were associated with reductions in ICU and hospital LOS in ICU patients overall. However, further research is needed for surgical patients and those with pneumonia.

## 1. Introduction

Prolonged hospitalisation, in particular prolonged admission to the Intensive Care Unit (ICU), is associated with multiple potential harms to patients. One extra day in hospital increases the probability of developing a hospital-acquired infection by as much as 1.37% [1]. Functional decline is seen in approximately half of patients admitted to the ICU [2]. Delirium affects up to 74% of patients admitted to the ICU [3]. Prolonged hospital and ICU admissions also result in excess financial costs. A German review calculated the daily cost of a non-ventilated admission to ICU to be €999 (95% confidence interval [CI] €924 - €1,074), and daily costs for ventilated patients to be €1,590 (95% CI €1,524 - €1,657) [4]. In the United States of America, the total annual costs for the five major hospital acquired infections amounts to $9.8 billion (95% CI $8.3-$11.5 billion) [5]. Safely reducing the length of time a patient spends in hospital and/or in the ICU is therefore of relevance to healthcare providers, patients, and policy makers.

Sleep disturbance is common among patients admitted to the ICU mainly due to noise, light, and frequent patient care activities [6]. Interference with the natural sleep cycle has detrimental effects on multiple body systems [7]. Sleep disturbance is associated with metabolic effects such as impaired glucose sensitivity [8], psychiatric effects such as depression [9], and is implicated as a risk factor for delirium although a causal link between sleep disruption and delirium has been difficult to establish [10].

Melatonin is a hormone with multiple physiological functions, including the promotion of sleep [11]. Melatonin receptor agonists (e.g., ramelteon and tasimelteon) are designed to act at the same receptors, namely the MT1 and MT2 melatonin receptors [12], but have a longer half-life and higher affinity for these receptors [13]. Exogenous melatonin and melatonin receptor agonists are commonly prescribed in the ICU to promote sleep, despite a dearth of evidence to support this practice [14,15]. While exogenous melatonin and melatonin receptor agonists are inexpensive and generally well tolerated, they are not inert treatments, and a small percentage of patients experience adverse effects such as headache, somnolence, palpitations, and abdominal pain [16]. To help clinicians decide whether potential benefits outweigh these harms, there is a need to determine whether melatonin supplementation influences ICU and/or hospital length of stay.

A recent systematic review and meta-analysis by Khaing and Nair [17] found that melatonin reduces the risk of delirium in patients admitted to the ICU by 34%. Zhang et al. [18] also found that exogenous melatonin and melatonin receptor agonists could improve sleep for patients admitted to the ICU and proposed that this may play an important role in decreasing the prevalence of delirium and shortening duration of ICU stay. Dziegielewski et al. [19] conducted a review which found that delirium adds on average 4.77 days to ICU length of stay and on average an additional $3,921 in ICU costs. Given that melatonin has been shown to reduce the risk of delirium, and delirium is associated with increased hospital and ICU length of stay, this systematic review and meta-analysis aims to identify and critically appraise the literature on the effect of exogenous melatonin or melatonin receptor agonists on ICU and/or hospital length of stay among adults admitted to the ICU. The primary objectives of this review were to investigate the relationship between exogenous melatonin or melatonin receptor agonists in the ICU, and ICU length of stay as well as the total hospital length of stay. As secondary objectives, this review aimed to examine the effect of the dose of exogenous melatonin or melatonin receptor agonist on the length of ICU and/or hospital stay, and to examine the relationship between exogenous melatonin or melatonin receptor agonist, ICU and/or hospital length of stay, and patient age.

## 2. Methods

This systematic review and meta-analysis was conducted in accordance with the *Cochrane Handbook for Systematic Reviews of Interventions* [20], and reported using the Preferred Reporting Items for Systematic Reviews and Meta-Analyses (PRISMA) checklist [21] (Table S1).

### 2.1 Eligibility criteria

A modified version of Richardson et al.’s [22] population, intervention, comparison, outcome (PICO) framework was used to include setting and study type. We included studies involving adult patients (≥18 years old) who received exogenous melatonin supplementation or treatment with a melatonin receptor agonist. Selected studies required a comparison group to those receiving melatonin supplementation or a melatonin receptor agonist. Length of stay in the ICU and/or total length of hospital stay following ICU admission were the outcome measures required. Studies had to be conducted in an ICU setting and only randomised control trials (RCTs) were included. Table S2 outlines the inclusion and exclusion criteria in full along with sample search terms.

### 2.2 Search strategy

First, a scoping search of the grey literature was conducted in the National Institute for Health and Care Excellence (NICE) [23], Health Information and Quality Authority (HIQA) [24], Health Service Executive (HSE) [25], World Health Organisation (WHO) [26], Google [27], and Google Scholar [28], to identify relevant and appropriate search terms. The following databases were then searched for relevant studies: PubMed, Cumulative Index to Nursing and Allied Health Literature (CINAHL), the Cochrane Library, Academic Search Complete, Embase, and Scopus. Trial registries were also searched including: ClinicalTrials.gov, the European Union Clinical Trials Registry and the International Clinical Trials Registry Platform. A search was also conducted in Google Scholar and the first 200 hits were screened.

Major subject headings were used as appropriate, and the search was customised for each database. The search was constructed from three key concepts namely: Melatonin, ICU, and length of stay. These concepts were combined using Boolean operators ‘OR’ and ‘AND’. The full search strategies for each database are included in Table S3.

The search was last conducted on the 11^th^ of November 2024. No database limits were applied to maximise the number of potential studies and reduce the risk of study selection bias.

### 2.3 Study selection

Studies identified from the search were imported to the web-based systematic review management software Covidence [29], where screening was conducted. Duplicates were automatically excluded by Covidence. Studies were screened by title and abstract by two reviewers and conflicts were resolved by a third reviewer. Full text screening was then performed by two reviewers and again, any conflicts were resolved by a third reviewer. The reasons for excluding studies at full text screening stage were recorded.

### 2.4 Data extraction

Data were extracted from the included studies and entered into a pre-formulated data extraction table (Table S4). Data extraction was completed by one researcher and a sample of extracted data was cross checked by a second reviewer for accuracy. Data extraction headings were decided following consultation with senior researchers and were agreed to be in line with the research objectives for this review.

### 2.5 Risk of bias, quality appraisal, and level of evidence assessment

Risk of bias assessment was conducted for each of the included studies using the Cochrane Risk of Bias 2 (RoB 2) tool [30]. One researcher completed risk of bias assessment and a second researcher selected a random sample of these assessments for cross-checking. Risk of bias assessment results were input into robvis [31], a web-based application designed for the visualisation of risk of bias assessments in a systematic review.

Each study also underwent quality appraisal. One researcher used the RCT items from the Mixed Method Appraisal Tool (MMAT) [32] to appraise the methodological quality for each study. A random sample was selected by a second researcher for evaluation.

The Grading of Recommendations Assessment, Development, and Evaluation (GRADE) system [33] was used to assess the certainty of evidence for outcomes assessed in this systematic review and meta-analysis. This method involves assessing the level of evidence across outcomes in five domains: risk of bias (across studies), inconsistency, indirectness, imprecision, and publication bias.

### 2.6 Data synthesis

The Cochrane Handbook guidance on undertaking meta-analyses was used to conduct meta-analyses of the data [34]. Quantitative analysis was performed in R (version 4.4.2) [35] using the packages “meta” (version 8.0–2) [36] and “metasens” (version 1.5–3) [37]. An inverse variance method was utilised for meta-analysis with disease type subgroups and was displayed using forest plots. Medians and interquartile ranges were converted to means and standard deviations (SD) [38]. Results were expressed as mean difference (MD) with 95% confidence intervals (CIs). Meta-regression was used for the secondary objectives to assess if the study-level characteristics of melatonin dosage and mean patient age impacted the standardized MD.

The degree of heterogeneity across studies was assessed using the I^2^ statistic with 95% CI. Statistically significant heterogeneity was defined as p-value ≤ 0.05. Prediction intervals were reported to allow for an estimated range within which future studies would be expected to occur and to avoid some of the ambiguity associated with I^2^ statistic [39,40]. A parametric bootstrap approach using a confidence distribution developed by Nagashima et al. was employed to calculate the prediction intervals [41]. The authors subsequently demonstrated that this method performs well despite high heterogeneity and low numbers of studies; producing accurate prediction intervals when the I^2^ statistics are in the range 7–99% and the number of studies is ≥ 3.

Results from meta-analyses were summarised using forest plots. Baujat plots were generated to illustrate the influence of each study on the pooled results and their contribution to overall heterogeneity. Funnel plots were used to indicate potential small study effects or publication bias, and the statistical significance of these effects was assessed using Egger’s test (i.e., a weighted linear regression between the treatment effects and standard errors of the studies) [42]. Bias correction was applied to the meta-analyses using the Duval & Tweedie trim and fill method [43], and the limit meta-analysis as proposed by Rücker et al. [44].

## 3. Results

### 3.1 Study selection

**Fig 1:**
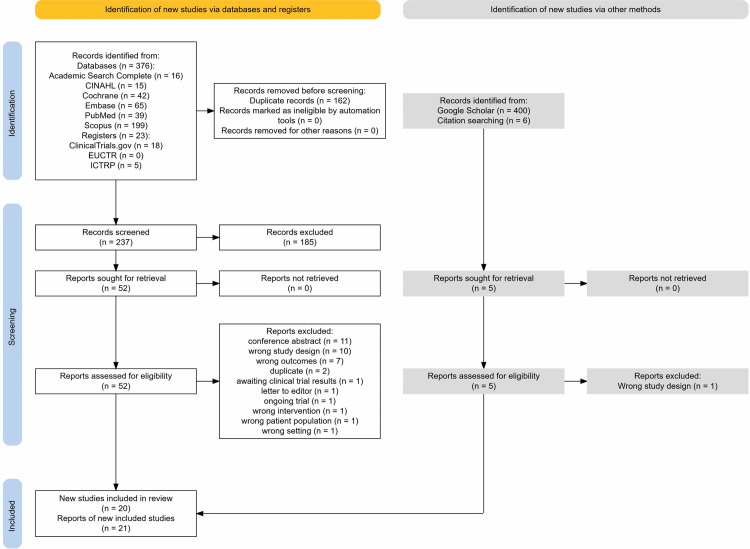
Study identification, screening and selection process [45].

A total of 376 records were identified from databases and 23 records from trial registers. After removal of 162 duplicates, 237 records were screened based on title and abstract. Full text screening was conducted for 57 records following exclusion of 180 irrelevant records. A further 400 records were identified from Google Scholar, and 6 records from citation searching records which were screened in the full text review and deemed eligible for data extraction. Of these, 5 records were assessed for eligibility based on full text review and 1 study was excluded at this stage. In total, 20 studies were included in this review (Fig 1).

### 3.2 Study characteristics

Twenty-one reports [46–66] were included in this systematic review presenting the findings of 20 unique studies (i.e., patient cohorts). The articles published by Dianatkhah et al. [50] and Sharifnia et al. [60] were reporting on the same patient cohort therefore, for data synthesis purposes they were treated as one study in this review. There was a wide geographical spread, with Iran being the country most frequently represented in the included studies (n = 8) [46,47,50,52,57,60,62,63,66]. Studies were published between 2011 [58] and 2024 [48]. Sample size ranged from 29 [55], to 847 participants [65]. The included RCTs comprised a total of 2,767 patients. Only two were multi-centre trials [45,46].

Ten studies [46,47,51–54,57,58,63,65] reported on the difference in both, ICU length of stay and total hospital length of stay between groups which received melatonin or a melatonin receptor agonist compared to a control group. Eight studies [48–50,56,59,60,62,64,66] reported on ICU length of stay alone, while two studies reported on hospital length of stay alone [55,61]. Only Dianatkhah et al. [50], Nishikimi et al. [59], and Sharifnia et al. [60] reported on ICU length of stay and/or hospital length of stay as a primary outcome.

Two studies used a melatonin receptor agonist as their intervention and both used ramelteon [53,59]. All other studies used exogenous melatonin as their intervention. There was considerable heterogeneity in dose and duration of melatonin supplementation across studies. For example, Vijayakumar et al. [64] gave 3 mg of melatonin at 9 pm daily for the duration of a patient’s ICU stay while Nickkholgh et al. [58] gave a single 50 mg/kg of body weight dose of melatonin in 250 ml of milk via nasogastric tube preoperatively. A sample summary of study characteristics is included in Table 1 below. A full description of study characteristics is outlined in the data extraction table (Table S4).

**Table 1:** Sample of key study characteristics (n = 20).

**Country**	Iran (n = 8) Australia (n = 2) India (n = 2) Brazil (n = 1) China (n = 1) Egypt (n = 1) Germany (n = 1) Italy (n = 1) Japan (n = 1) Spain (n = 1) USA (n = 1)
**Setting**	Single centre (n = 18) Multicentre (n = 2)
**Sample size (Range)**	29–847
**Relevant outcome**	ICU and hospital length of stay (n = 10) ICU length of stay only (n = 8) Hospital length of stay only (n = 2)
**Intervention**	Melatonin (n = 18) Ramelteon (n = 2)

### 3.3 Risk of bias assessment

**Fig 2:**
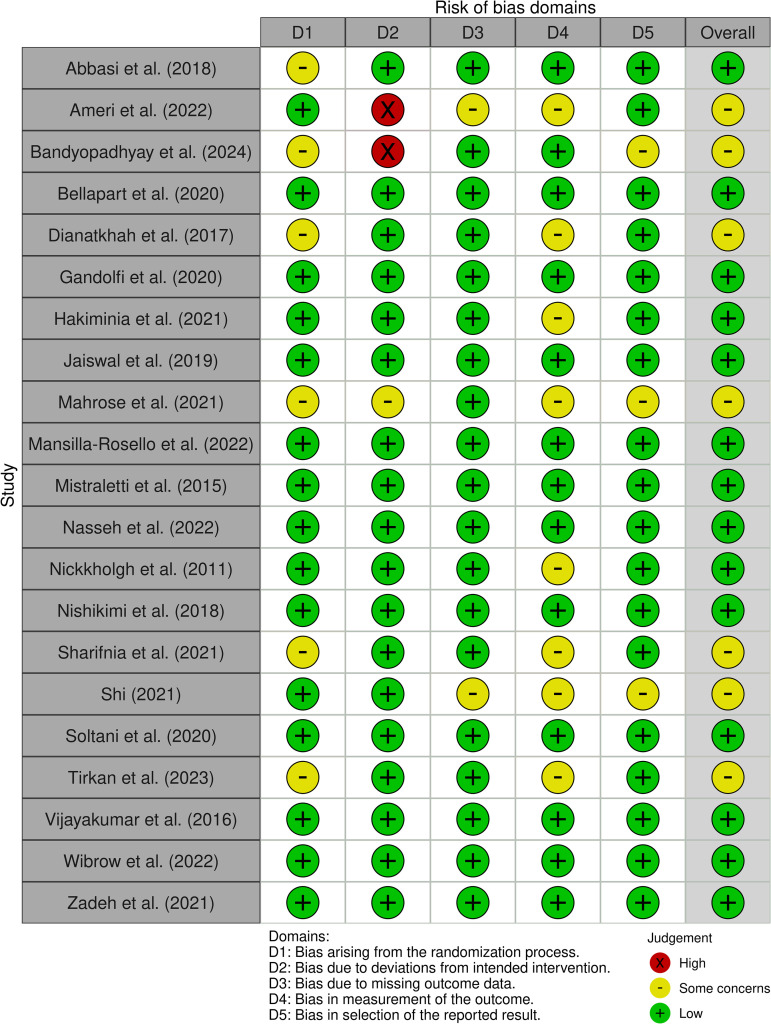
“Traffic light” plot of the domain-level risk of bias judgements for RCTs.

**Fig 3:**
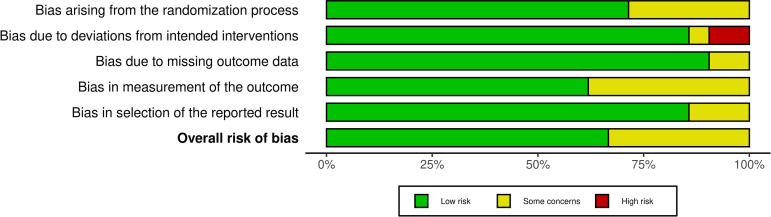
Weighted bar plots of the distribution of risk of bias judgements within each bias domain for RCTs.

Fourteen of the included RCTs had a low overall risk of bias [46,49,51–53,55–59,62,64–66]. The remaining seven had some concerns [47,48,50,54,60,61,63]. None of the RCTs were judged to have a high risk of bias. The studies by Dianatkhah et al. [50] and Sharifnia et al. [60] were assessed separately given they were reporting on different primary outcomes. Fig 2 below illustrates a “traffic light” plot of the domain-level judgements for each RCT generated using robvis [31], while Fig 3 demonstrates weighted bar plots of the distribution of risk of bias judgements within each bias domain for each RCT generated using robvis [31].

### 3.4 Quality appraisal

Eight studies met all MMAT domains [46,52,53,56,58,59,62,64]. It was not clear if randomisation was performed appropriately in three studies [54,55,57]. Groups were comparable at baseline in all but two studies [48,49]. Three studies did not contain complete outcome data [55,61,65]. This was unclear in one study since links provided for supplemental data were not functioning [45]. All but three studies [50,54,60,63] blinded outcome assessors to the intervention but it was unclear whether this was done correctly in one further study [66]. Three studies [47–49] did not adhere fully to the assigned intervention (Table 2).

**Table 2:** Quality assessment of the included studies (n = 20) using the Mixed Method Appraisal Tool [32].

Author(s) & year	Quality appraisal items						
	Clear research questions/aims	Data collected address research question/aims	Randomisation appropriately performed	Groups comparable at baseline	There are complete outcome data	Outcome assessors blinded to the intervention	Participants adhered to the assigned intervention
Abbasi et al. (2018)	Y	Y	Y	Y	Y	Y	Y
Ameri et al. (2022)	Y	Y	Y	Y	Y	Y	N
Bandyopadhyay et al. (2024)	Y	Y	Y	CT	Y	Y	N
Bellapart et al. (2020)	Y	Y	Y	CT	Y	Y	N
Dianatkhah et al. (2017)^*^	Y	Y	Y	Y	Y	N	Y
Gandolfi et al. (2020)	Y	Y	Y	Y	CT	Y	Y
Hakiminia et al. (2021)	Y	Y	Y	Y	Y	Y	Y
Jaiswal et al et al. (2019)	Y	Y	Y	Y	Y	Y	Y
Mahrose et al. (2021)	Y	Y	CT	Y	Y	N	Y
Mansilla-Rosello et al. (2022)	Y	Y	CT	Y	N	Y	Y
Mistraletti et al. (2015)	Y	Y	Y	Y	Y	Y	Y
Nasseh et al. (2022)	Y	Y	CT	Y	Y	Y	Y
Nickkholgh et al. (2011)	Y	Y	Y	Y	Y	Y	Y
Nishikimi et al. (2018)	Y	Y	Y	Y	Y	Y	Y
Shi (2021)	Y	Y	Y	Y	N	Y	Y
Soltani et al. (2020)	Y	Y	Y	Y	Y	Y	Y
Tirkan et al. (2023)	Y	Y	Y	Y	Y	N	Y
Vijayakumar et al. (2016)	Y	Y	Y	Y	Y	Y	Y
Wibrow et al. (2022)	Y	Y	Y	Y	N	Y	Y
Zadeh et al. (2021)	Y	Y	Y	Y	Y	CT	Y

*Also reported in Sharifnia et al. [60].

**Abbreviations:** CT=Can’t Tell, N=No, Y=Yes

### 3.5 Synthesis of results

As aforementioned, Dianatkhah et al. [50] and Sharifnia et al. [60] have been treated as one study for the purpose of meta-analysis as they involved the same study population. Two meta-analyses were conducted: one to address ICU length of stay and a second to address hospital length of stay. There were insufficient data reported to conduct meta-analyses on the effect of exogenous melatonin or melatonin receptor agonist dose on hospital and/or ICU length of stay, neither were there sufficient data for a meta-analysis to address the relationship between exogenous melatonin or melatonin receptor agonist, hospital and/or ICU length of stay, and patient age. Findings from individual studies are presented in Table S4. For simplicity, “melatonin receptor agonist” is replaced with “ramelteon” hereafter since this was the only melatonin receptor agonist identified in the selected studies.

#### 3.5.1 Relationship between exogenous melatonin or ramelteon treatment in the intensive care unit, and intensive care unit length of stay.

**Fig 4:**
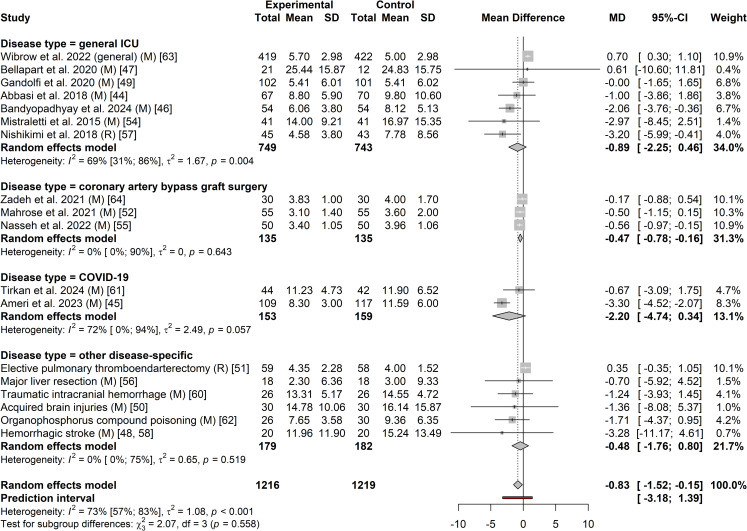
Forest plot of the effect of exogenous melatonin or ramelteon on ICU length of stay with disease-specific subgroups.

**Fig 5:**
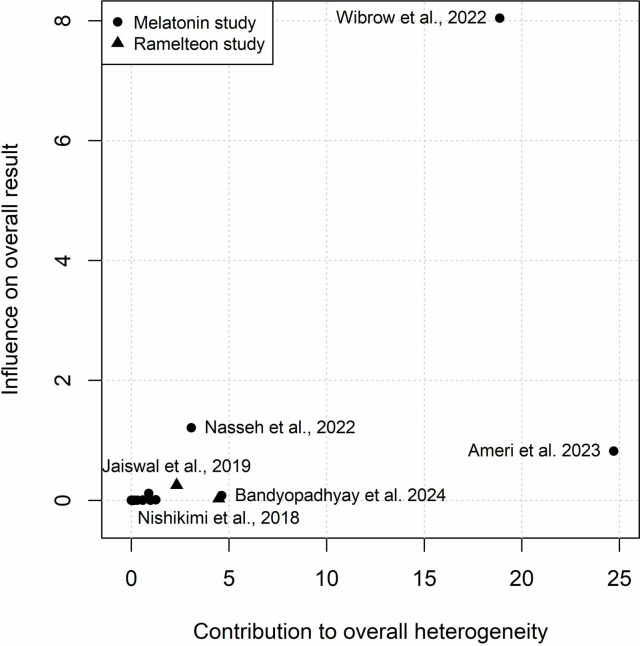
Baujat plot for ICU length of stay illustrating the influence of each study on the pooled results and their contribution to overall heterogeneity.

**Fig 6:**
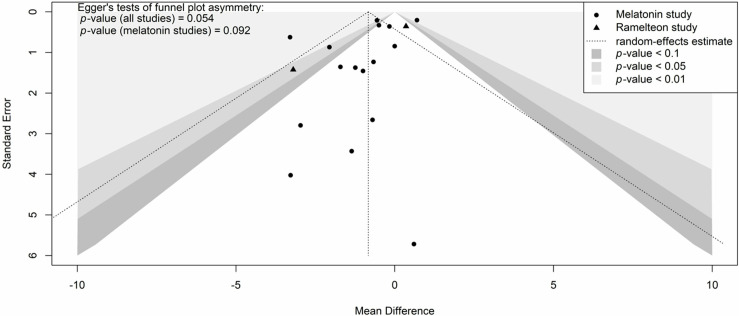
Contour-enhanced funnel plot of the effect of exogenous melatonin or ramelteon on ICU length of stay with disease-specific subgroups.

Eighteen studies involving 2,435 participants were included in the meta-analysis on the effect of exogenous melatonin or ramelteon on ICU length of stay [46–54,56–60,62–66]. Sixteen [46–52,54,56–58,60,62–66] studies reported on the effect of melatonin on ICU length of stay and two [53,59] reported on ramelteon’s effect on ICU length of stay. There was high heterogeneity across groups (*I*^2^ = 73% with the prediction interval indicating that based on current results we can be 95% certain that the MD in the next new study would be between −3.18 and 1.39 days (Fig 4). The high levels of heterogeneity were mainly the result of two statistical outliers with the Pro-MEDIC trial [65] and Ameri et al. 2023 [47] (Fig 5). As indicated in Table S5 these outliers impact the results for general ICU patients. The MD for melatonin studies without these outliers (Table S6; I^2^ = 0%) was −0.55 days (95% CI: −0.84 to −0.25; *p*-value < 0.001). Positive results were also seen for patients that had coronary artery bypass graft surgery with a MD of −0.47 days (95% CI: −0.78 to −0.16; *p*-value = 0.003) (Fig 4). Visual representation of potential publication bias using a funnel plot (Fig 6) appeared to demonstrate publication bias. While this did not reach statistical significance as per Egger’s test (T = −2.08, *p* = 0.054), when bias-correction was applied to the meta-analysis (Table S7) it resulted in a statistically insignificant MD (*p*-values > 0.05).

#### 3.5.2 Relationship between exogenous melatonin or ramelteon treatment in the intensive care unit, and total hospital length of stay.

**Fig 7:**
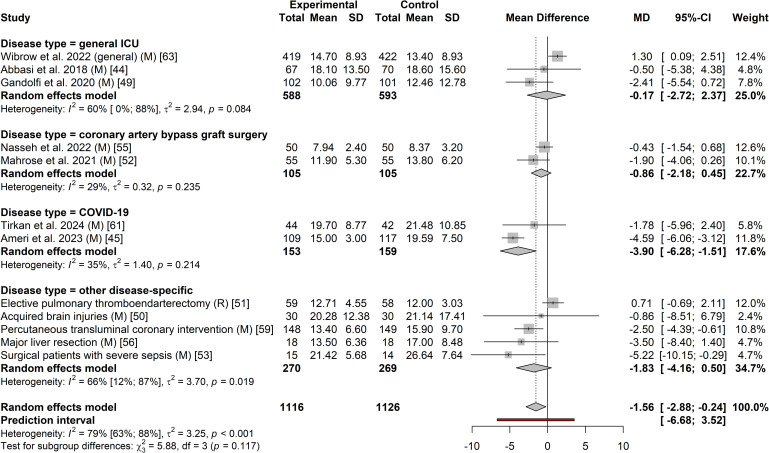
Forest plot of the effect of exogenous melatonin or ramelteon on hospital length of stay.

**Fig 8:**
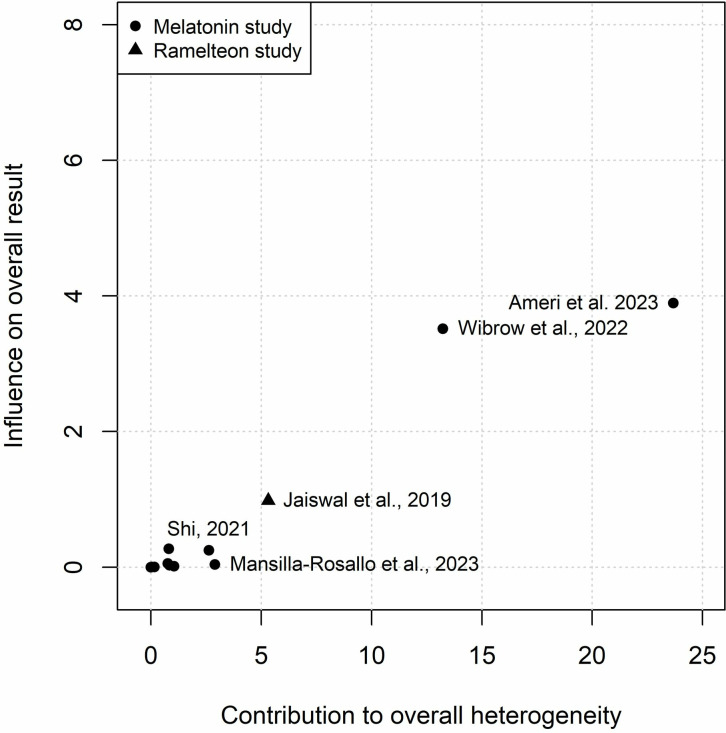
Baujat plot for hospital length of stay illustrating the influence of each study on the pooled results and their contribution to overall heterogeneity.

**Fig 9:**
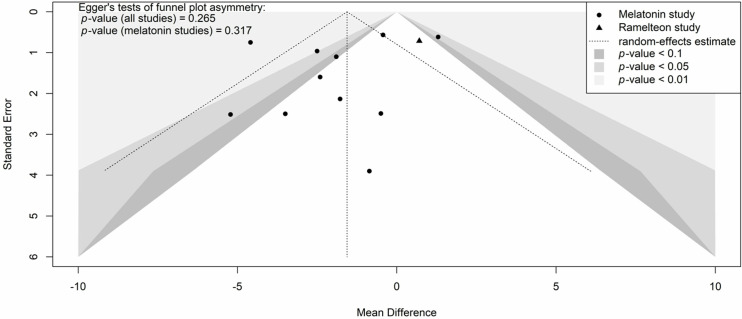
Contour-enhanced funnel plot of the effect of exogenous melatonin or ramelteon on hospital length of stay.

Twelve studies involving 2,242 participants were included in the meta-analysis on the effect of exogenous melatonin or ramelteon on total hospital length of stay [46,47,51–55,57,58,61,63,65]. Eleven studies [46,47,51,52,54,55,57,58,61,63,65] used melatonin while only one study used ramelteon [53]. Heterogeneity was also high for hospital length of stay (*I*^2^ = 79%with a 95% prediction interval indicates that the MD in the next new study would likely be between −6.68 and 3.52 days (Fig 7). The heterogeneity was mostly the result of two statistical outliers Pro-MEDIC trail [65] and Ameri et al. 2023 [47] (Fig 8, Table S5). The MD for melatonin studies without these outliers (Table S6; I^2^ < 1%) was −1.71 days (95% CI: −2.78 to −0.63; *p*-value = 0.002). Positive results were also seen for patients that had COVID-19 with a MD of −3.90 days (95% CI: −6.28 to −1.51; p-value = 0.001) (Fig 7). Egger’s test was not statistically significant (T = −1.18, *p* = 0.265) for publication bias however when bias-correction was applied to the meta-analysis (Table S7) it resulted in a statistically insignificant MD (*p*-values > 0.05). A visual representation of potential publication bias is demonstrated in the funnel plot below (Fig 9).

#### 3.5.3 Effect of the dose of melatonin or ramelteon treatment on the length of ICU and/or hospital stay.

None of the included studies examined whether the dose of melatonin or melatonin receptor agonist affected length of stay in ICU and/or hospital stay. There was considerable heterogeneity between the dose of melatonin received by patients with 3 mg once daily being on the lower end of the scale but the most frequently studied dose [46,48,57,61,62,64,66], while higher doses were as much as 30 mg as a single dose [50,60], or 60 mg as an infusion over 24 hours [55] or 50 mg/kg of body weight [58]. Duration of treatment with exogenous melatonin ranged from a single pre-operative dose [58] to 14 consecutive nights [65], although in this study participants stopped receiving melatonin if they were discharged from ICU prior to day 14. Route of administration for exogenous melatonin was oral or via nasogastric tube in all but one study which instead gave it as an infusion over 24 hours [55]. In both ramelteon studies [53,59], the standard 8 mg dose of ramelteon was given, either orally or via nasogastric tube. Using meta-regression analysis for studies with melatonin (3 mg to 60 mg daily) there was no significant association between the MD and melatonin dosage for either ICU length of stay (15 studies, *p*-value = 0.708) or hospital length of stay (10 studies, *p*-value = 0.340).

#### 3.5.4 Relationship between melatonin or ramelteon treatment, ICU and/or hospital length of stay, and patient age.

None of the included studies examined the relationship between exogenous melatonin or ramelteon treatment, ICU and/or hospital length of stay, and patient age. The mean age in each study ranged between 34.90 years [48] and 66.55 years [54]. Using meta-regression analysis for studies with melatonin (3 mg to 60 mg daily) there was no significant association between the MD and the patients’ mean age for either ICU length of stay (15 studies, *p*-value = 0.371) or hospital length of stay (10 studies, *p*-value = 0.709).

### 3.6 Level of evidence assessment

The level of evidence was found to be low with regards to both primary outcomes when assessed using the GRADE [33] system. Despite limiting studies to RCTs, the level of evidence for both primary outcomes was downgraded due to inconsistency and imprecision.

Regarding ICU length of stay, the downgrade for inconsistency was due to high heterogeneity across studies and a prediction interval crossing zero. The downgrade for imprecision was based on a 95% CI close to the null and a prediction interval which included potential harm. In addition, ten of the 18 studies reported medians instead of means which may have reduced the accuracy of the meta-analysis estimates of MD. Publication bias was considered due to visual funnel plot asymmetry, and while Egger’s test did not reach statistical significance a downgrade was still applied since it resulted in statistically insignificant results for ICU length of stay when incorporated into the meta-analysis (trim and fill and limit methods).

For hospital length of stay, inconsistency was again due to high heterogeneity and a wide prediction interval. The level of evidence was downgraded due to imprecision as the CI crossed zero and the prediction interval again included potential harm and five of the 12 studies reported medians. Bias-corrected meta-analysis methods again produced statistically insignificant results.

As outlined in the data synthesis section, the secondary outcomes, were assessed by taking the mean age and dosage from each study and had the same limitations as the overall analysis. A level of evidence assessment is outlined below in Table 3.

**Table 3:** Level of evidence assessment per review outcome.

Outcomes	Number of participants (Studies)	Initial certainty	Risk of bias	Inconsistency	Indirectness	Imprecision	Publication bias	Overall quality (GRADE)
ICU Length of stay	2,435 (18 studies)	High (RCTs)	—	Downgrade 1	—	Downgrade 1	Downgrade 1	+OOO Very low
Hospital length of stay	2,242 (12 studies)	High (RCTs)	—	Downgrade 1	—	Downgrade 1	Downgrade 1	+OOO Very low
ICU Length of stay coronary artery bypass	2,435 (18 studies)	High (RCTs)	—	—	—	Downgrade 1	Downgrade 1	++OO Low

**Abbreviations:** ICU = Intensive Care Unit, RCT = Randomised Control Trial

For the disease type sub-analyses the evidence for coronary artery bypass graft surgery and ICU length of stay was consistent (I^2^ = 0%) although it is limited to only three studies.

## 4. Discussion

### 4.1 Summary of findings

To the best of our knowledge, this is the first systematic review and meta-analysis to examine the effect of exogenous melatonin and melatonin receptor agonists on ICU and hospital length of stay. Previous systematic reviews and meta-analyses have examined the effect of melatonin on the promotion of sleep in adult patients in the ICU [14], and the effect of melatonin on delirium prevention in hospitalised patients [17].

This review was limited to RCTs. A total of 20 studies were included. Eighteen studies with a total of 2,435 participants were included in the meta-analysis of exogenous melatonin or melatonin receptor agonists’ effect on ICU length of stay while twelve studies with a total of 2,242 participants were included in the meta-analysis to address the effect on hospital length of stay. The only melatonin receptor agonist identified in the included studies was ramelteon. Ramelteon is licensed for the treatment of insomnia by the FDA [67] and exerts its effects by binding and activating MT1 and MT2 melatonin receptors [12].

Significant differences were observed in the route, frequency, and timing of melatonin administration in this analysis. This is consistent with previous reviews examining the effects of melatonin on other outcomes [68,69]. The optimum dose of melatonin is unclear. A systematic review by Vural et al. [70] advised using the lowest dose of melatonin possible in older patients to avoid prolonged, supra-physiological blood levels. We were unable to conduct analysis on the effect of melatonin or melatonin receptor agonists dosage on length of stay due to the heterogeneity between studies and lack of reported data. Similarly, we could not assess whether effects on length of stay differed across patient age groups.

Our systematic review and meta-analysis provided a very low certainty of evidence a reduction in either ICU or hospital length of stay for adult patients who received either exogenous melatonin or a melatonin receptor agonist. This was cause by inconsistent results and a lack of statistical significance when the meta-analysis is adjusted for potential publication bias (small study effects). However, reductions were observed in ICU stay for patients that had coronary artery bypass graft surgery (3 studies [54,57,66]) and in hospital stay for patient with COVID-19 (2 studies [47,63]). It is worth mentioning that this review was initially conducted in February 2023 but not published at that time. That initial review did not reach statistical significance for either ICU or hospital length of stay. We repeated the review in November 2024 due to the publication of relevant studies in the interim.

### 4.2 Implications

This systematic review and meta-analysis use of melatonin and melatonin receptor agonists to reduce ICU and hospital length of stay in those with coronary artery bypass graft surgery and COVID-19 and these findings warrant further research in surgical and pneumonia patients. Previous reviews have shown some benefits, especially on the incidence of delirium [69], and in particular post-operative delirium [71], when exogenous melatonin or melatonin receptor agonists are prescribed to patients admitted to the ICU. Delirium is a key factor which has been demonstrated to prolong length of stay [72]. Much of the benefit seen in this study may be due to lower rates of delirium for patients receiving melatonin or melatonin receptor agonists.

Safely reducing ICU and hospital length of stay has knock on effects on the cost of healthcare. While we did not specifically address cost in this review, it stands to reason that reducing the length of time a patient spends in ICU and subsequently in hospital, will have a knock on cost saving effect for the healthcare system. This is an important factor to consider in an era of increasing healthcare costs.

The dose of melatonin varied significantly across studies. None of the studies compared different doses of melatonin and the rationale for their chosen dose was not often clear. Eight studies used a dose of 4 mg or less per day [46,48,57,61,62,64–66] but within this group there was heterogeneity between the timing of doses. The optimum timing for melatonin dosing again remains unclear. A meta-regression analysis failed to find any statistically significant association between dosage and MD effect size. The COVID-19 patient study with a large effect size used a dosage of 5 mg [47], and the studies with coronary artery bypass graft surgery patients used 3–5 mg [54,57,66]. These findings suggest that 3–5 mg per day would be sufficient although even very large dosages of 50 mg/kg of body weight were found to be safe [58].

Ramelteon demonstrated similar effects as exogenous melatonin but was only used in two of the included studies [53,59]. Ramelteon has been associated with reduced rates of delirium in older patients [73]. This may warrant further studies to investigate the effect of ramelteon on ICU and/or hospital length of stay. These studies are unlikely to be forthcoming as the product has not been licenced by the European Medicines Agency [74].

### 4.3 Limitations

Missing data was the largest limiting factor in this review. Half the studies (n = 10) reported median instead of mean values which may have led to less precise meta-analysis results when mean values were estimated. In addition, there was no rich data for the secondary objectives with no studies reporting on differences in length of stay across different age groups, and a meta-regression using the mean age of the participants in the study was considered instead.

Only three studies [50,59,60] reported the effects on ICU or hospital length of stay as primary outcomes and selective reporting of positive findings in small studies may have been present. Although Egger’s test did not show any statistically significant publication bias, this must be interpreted with the knowledge that the test itself has certain limitations including a dependence on *p*-values, the ‘file-drawer problem’ [75], and limited power when the number of selected studies is small. Supplementing this with additional bias-corrected meta-analysis methods revealed that the reduction in length of stay for both the ICU and hospital stay were not statistically significant if corrected for the asymmetry in small study effects. It is worth noting that the largest included RCT (Pro-MEDIC [65]) including 12 Australian ICUs reported no statistically significant differences with a trend in the direction of harm for both ICU length of stay (median: 5 vs 5 days, p = 0.135) and hospital length of stay (median: 14 vs 12 days, p = 0816) [65].

Heterogeneity across studies is a limitation when trying to identify an overall effect size. We have focused on reporting prediction intervals which are valid in this context. In addition, subgroup analysis by disease type was carried out to combine result for more clinically homogenous patients. Promising finding were observed for patients who received coronary artery bypass graft surgery although this was based on just three studies. Overall, it was found that the main statistical outliers were the large Pro-MEDIC [65] (discussed above) and a COVID-19 study which found very significant improvements. Further research is needed for surgical and pneumonia patients. Nevertheless, the degree of heterogeneity may have been problematic for addressing our secondary outcomes of age and dosage differences which we limited to melatonin studies using 3 mg to 60 mg daily.

## 5. Conclusion

In conclusion, the present systematic review and meta-analysis demonstrated very low certainty regarding a reduction in ICU and hospital length of stay for adults patients admitted to the ICU who received melatonin or melatonin receptor agonists. Promising findings were observed for patients that had coronary artery bypass graft surgery or COVID-19 induced pneumonia on 3 mg to 5 mg daily. Regarding the secondary objectives, we did not identify any statistically significant differences according to the participants’ mean age or dosage in melatonin studies. Only two ramelteon studies were identified with inconsistent results. Further research is needed to assess the association between melatonin and length of stay for surgical and pneumonia ICU patients.

## Supporting information

Table S1 PRISMA 2020 Checklist. (DOCX)

Table S2 Review eligibility criteria and sample search terms. (DOCX)

Table S3 Complete search strategy. (DOCX)

Table S4 Data extraction table. (DOCX)

Table S5 Breakdown of meta-analysis results by disease group with the two statistical outliers. (DOCX)

Table S6 Breakdown of meta-analysis results by medication type with the two statistical outliers. (DOCX)

Table S7 Bias-corrected meta-results results. (DOCX)
