# Supplementary material for: The effect of exogenous melatonin and melatonin receptor agonists on intensive care unit and hospital length of stay: A systematic review and meta-analysis
Source: PLoS One. 2025 Sep 8;20(9):e0332031. doi: 10.1371/journal.pone.0332031 (PMC12416736; doi:10.1371/journal.pone.0332031)
Supplement: Table S2 — (DOCX) [file pone.0332031.s002.docx]

**Supplementary Table S2.** Review eligibility criteria and sample search terms

|  | **Inclusion Criteria** | **Exclusion Criteria** | **Search Terms^*^** |
| --- | --- | --- | --- |
| Population | Adult patients (≥18 years old) | Children (<18 years old) | None used |
| Intervention | Exogenous melatonin supplementation or treatment with a melatonin receptor agonist (e.g. ramelteon) | No exogenous melatonin supplementation or melatonin receptor agonist | “Melatonin supplementation”: “melatonin”; “melatonin admin*”;“melatonin dos*”; “melatonin treat*” |
| Comparison | Selected studies must include a comparison group to those receiving melatonin supplementation | No comparison group included | None used |
| Outcome Measure | Length of stay in ICU or total length of hospital stay following ICU admission | All other outcome measures not related to ICU/hospital length of stay | “Length of stay”: “hospital stay”; “hospitali*ation”; “LOS”; “duration of hospitali*tion”; “day* hospitali*ed”; “day* in hospital”; “duration of stay”; “time to discharge” |
| Setting | Studies conducted in the ICU setting | Studies conducted outside of an ICU setting | “ICU”: “ICUs”; “ITU”; “ITUs”; “Intensive Care Unit*”; “Intensive Therap*”; “Critical Care Unit*” |
| Study Design | Randomised control trials | Observational studies, qualitative research, case series, case reports, editorials, book chapters, theses, dissertations, conference proceedings. | None used |

*Sample search terms included in this table. The search strategy was customised for each of the reviewed databases and is included in Supplementary Table S3.
