# Supplementary material for: The effect of exogenous melatonin and melatonin receptor agonists on intensive care unit and hospital length of stay: A systematic review and meta-analysis
Source: PLoS One. 2025 Sep 8;20(9):e0332031. doi: 10.1371/journal.pone.0332031 (PMC12416736; doi:10.1371/journal.pone.0332031)
Supplement: Table S3 — (DOCX) [file pone.0332031.s003.docx]

## Supplementary Table S3. Complete search strategy

| **Database** | **Search Strings** | | | **Number of Hits** |
| --- | --- | --- | --- | --- |
| PubMed | **Key Search Terms 1:** |  |  |  |
|  | **melatonin[MeSH Terms]** |  |  | 22,802 |
|  | **"melatonin"[Title/Abstract]** |  |  | 29,009 |
|  |  | **(melatonin[MeSH Terms]) OR ("melatonin"[Title/Abstract])** |  | 30,777 |
|  | **Key Search Terms 2:** |  |  |  |
|  | **intensive care unit[MeSH Terms]** |  |  | 103,623 |
|  | **“ICU”[Title/Abstract]** |  |  | 80,530 |
|  | **"intensive care"[Title/Abstract]** |  |  | 187,775 |
|  | **“ITU”[Title/Abstract]** |  |  | 1,007 |
|  | **"intensive therap*"[Title/Abstract]** |  |  | 5,925 |
|  | **"critical care unit*"[Title/Abstract]** |  |  | 4,033 |
|  | **"critical care"[MeSH Terms]** |  |  | 65,699 |
|  |  | **((((((intensive care unit[MeSH Terms]) OR ("ICU"[Title/Abstract])) OR ("intensive care"[Title/Abstract])) OR ("ITU"[Title/Abstract])) OR ("intensive therap*"[Title/Abstract])) OR ("critical care unit*"[Title/Abstract])) OR (critical care[MeSH Terms])** |  | 283,018 |
|  | **Key Search Terms 3:** | | | |
|  | **length of stay[MeSH Terms]** |  |  | 101,482 |
|  | **LOS[Title/Abstract]** |  |  | 40,834 |
|  | **"hospital stay"[Title/Abstract]** |  |  | 95,641 |
|  | **"hospital day*"[Title/Abstract]** |  |  | 6,756 |
|  |  | **(((length of stay[MeSH Terms]) OR ("LOS"[Title/Abstract])) OR ("hospital stay"[Title/Abstract])) OR ("hospital day*"[Title/Abstract])** |  | 200,646 |
|  | **Final PubMed Search:** | | | |
|  |  |  | **(((melatonin[MeSH Terms]) OR ("melatonin"[Title/Abstract])) AND (((((((intensive care unit[MeSH Terms]) OR ("ICU"[Title/Abstract])) OR ("intensive care"[Title/Abstract])) OR ("ITU"[Title/Abstract])) OR ("intensive therap*"[Title/Abstract])) OR ("critical care unit*"[Title/Abstract])) OR (critical care[MeSH Terms]))) AND ((((length of stay[MeSH Terms]) OR ("LOS"[Title/Abstract])) OR ("hospital stay"[Title/Abstract])) OR ("hospital day*"[Title/Abstract]))** | 39 |
| CINAHL | **Key Search Terms 1:** | | | |
|  | **S1: (MH “melatonin”)** |  |  | 3,079 |
|  | S2: TI "melatonin" OR AB "melatonin" |  |  | 3,104 |
|  |  | S3: S1 OR S2 |  | 4,140 |
|  | **Key Search Terms 2:** | | | |
|  | **S4:** (MH "Intensive Care Units") |  |  | 45,079 |
|  | **S5:** (MH "Critical Care") |  |  | 25,764 |
|  | **S6:** TI "ICU" OR AB "ICU" |  |  | 36,915 |
|  | **S7:** TI “ITU” OR AB “ITU” |  |  | 348 |
|  | **S8: TI “intensive therap*” OR AB “intensive therap*”** |  |  | 1,617 |
|  | **S9: TI “critical care unit*” OR AB “critical care unit*”** |  |  | 2,690 |
|  |  | S10: S4 OR S5 OR S6 OR S7 OR S8 OR S9 |  | 82,572 |
|  | **Key Search Terms 3:** | | | |
|  | **S11:** (MH "Length of Stay") |  |  | 49,935 |
|  | **S12:** TI “LOS” OR AB “LOS” |  |  | 51,401 |
|  | **S13:** TI "hospital stay" OR AB "hospital stay" |  |  | 25,386 |
|  | **S14:** TI "hospital day*" OR AB "hospital day*" |  |  | 2,131 |
|  |  | S15: S11 OR S12 OR S13 OR S14 |  | 111,494 |
|  | **Final CINAHL Search:** | | | |
|  |  |  | S16: S3 AND S10 AND S15 | **15** |
| Cochrane Library | **Key Search Term 1:** | | | |
|  | **#1: MeSH descriptor: [Melatonin] explode all trees** |  |  | 1,481 |
|  | **#2: ("melatonin"):ti,ab,kw** |  |  | 3,511 |
|  |  | #3: #1 OR # 2 |  | 3,511 |
|  | **Key Search Terms 2:** | | | |
|  | **#4: MeSH descriptor: [Intensive Care Units] explode all trees** |  |  | 4,916 |
|  | **#5: MeSH descriptor: [Critical Care] explode all trees** |  |  | 2,632 |
|  | **#6: (“ICU”):ti,ab,kw** |  |  | 16,422 |
|  | **#7: (“ITU”):ti,ab,kw** |  |  | 1,791 |
|  | **#8: ("intensive therapy unit*"):ti,ab,kw** |  |  | 56 |
|  | **#9: ("critical care unit*”):ti,ab,kw** |  |  | 261 |
|  |  | #10: #4 OR #5 OR #6 OR #7 OR #8 OR #9 |  | 22,867 |
|  | **Key Search Terms 3:** | | | |
|  | **#11: MeSH descriptor: [Length of Stay] explode all trees** |  |  | 8,446 |
|  | **#12: ("LOS"):ti,ab,kw** |  |  | 14,663 |
|  | **#13: ("hospital stay”):ti,ab,kw** |  |  | 23,151 |
|  | **#14: ("hospital day*”):ti,ab,kw** |  |  | 277 |
|  |  | #15: #11 OR #12 OR #13 OR #14 |  | 41,384 |
|  | **Final Cochrane Library Search:** | | | |
|  |  |  | **#16: #3 AND #10 AND #15** | **42** |
| Academic Search Complete | **Key Search Terms 1:** | | | |
|  | **S1:** DE "MELATONIN" |  |  | 12,666 |
|  | **S2:** TI "melatonin" OR AB "melatonin" |  |  | 15,795 |
|  |  | S3: S1 OR S2 |  | 16,445 |
|  | **Key Search Terms 2:** | | | |
|  | **S4:** DE "INTENSIVE care units" |  |  | 38,038 |
|  | **S5: IT “ICU” OR AB “ICU”** |  |  | 43,932 |
|  | **S6:** TI “ITU” OR AB “ITU” |  |  | 3,167 |
|  | **S7:** TI "intensive therap*" OR AB "intensive therap*" |  |  | 1,919 |
|  | **S8: TI “critical care unit*” OR AB “critical care unit*”** |  |  | 1,922 |
|  |  | S9: S4 OR S5 OR S6 OR S7 OR S8 |  | 70,145 |
|  | **Key Search Terms 3:** | | | |
|  | **S10:** DE "LENGTH of stay in hospitals" |  |  | 21,874 |
|  | **S11:** TI “LOS” OR AB “LOS” |  |  | 454,278 |
|  | **S12: TI “hospital stay” OR AB “hospital stay”** |  |  | 42,735 |
|  | **S13: TI “hospital day*” OR AB “hospital day*”** |  |  | 2,540 |
|  |  | S14: S10 OR S11 OR S12 OR S13 |  | 508,204 |
|  | **Final Academic Search Complete Search:** | | | |
|  |  |  | **S15:** S3 AND S9 AND S14 | **16** |
| Embase | **Key Search Terms 1:** | | | |
|  | **#1: ‘melatonin’:ti,ab,kw** |  |  | 36,419 |
|  | **Key Search Terms 2:** | | | |
|  | **#2: ‘intensive care’:ti,ab,kw** |  |  | 277,333 |
|  | **#3: ‘icu’:ti,ab,kw** |  |  | 159,748 |
|  | **#4: ‘critical care unit*’:ti,ab,kw** |  |  | 6,955 |
|  | **#5: ‘intensive therap*’:ti,ab,kw** |  |  | 9,358 |
|  | **#6: ‘itu’:ti,ab,kw** |  |  | 3,069 |
|  |  | #7: #2 OR #3 OR #4 OR #5 OR #6 |  | 368,031 |
|  | **Key Search Terms 3:** |  |  |  |
|  | **#8: ‘length of stay’:ti,ab,kw** |  |  | 142,766 |
|  | **#9: ‘hospitalization’:ti,ab,kw** |  |  | 276,085 |
|  | **#10: ‘los’:ti,ab,kw** |  |  | 73,718 |
|  | **#11: ‘hospital day*’:ti,ab,kw** |  |  | 13,819 |
|  |  | #12: #8 OR #9 OR #10 OR #11 |  | 446,619 |
|  | **Final Embase Search:** | | | |
|  |  |  | **#13: #1 AND #7 AND #12** | **65** |
| Scopus | **Key Search Terms 1:** | | | |
|  | TITLE-ABS-KEY ( melatonin ) |  |  | 46,721 |
|  | **Key Search Terms 2:** | | | |
|  | **TITLE-ABS-KEY ( “intensive care unit* )** |  |  | 282,561 |
|  | **TITLE-ABS-KEY ( “intensive therap* )** |  |  | 7,572 |
|  | **TITLE-ABS-KEY ( “icu” )** |  |  | 93,786 |
|  | **TITLE-ABS-KEY ( “itu” )** |  |  | 12,948 |
|  | **TITLE-ABS-KEY ( “critical care unit*” )** |  |  | 4,887 |
|  |  | ( TITLE-ABS-KEY ( "critical care unit*" ) ) OR ( TITLE-ABS-KEY ( "intensive care unit*" ) ) OR ( TITLE-ABS-KEY ( "intensive therap*" ) ) OR ( TITLE-ABS-KEY ( icu ) ) OR ( TITLE-ABS-KEY ( itu ) ) |  | 322,371 |
|  | **Key Search Terms 3:** | | | |
|  | **TITLE-ABS-KEY**(**"length of stay"**) |  |  | 236,925 |
|  | **TITLE-ABS-KEY**(**"los"**) |  |  | 229,223 |
|  | **TITLE-ABS-KEY**(**"hospitali*ation"**) |  |  | 507,023 |
|  | **TITLE-ABS-KEY**(**"hospital stay"**) |  |  | 116,476 |
|  | **TITLE-ABS-KEY**(**"hospital day*"**) |  |  | 7,803 |
|  |  | ( TITLE-ABS-KEY ( "length of stay" ) )  OR  ( TITLE-ABS-KEY ( "los" ) )  OR  ( TITLE-ABS-KEY ( "hospitali*ation" ) )  OR  ( TITLE-ABS-KEY ( "hospital stay" ) )  OR  ( TITLE-ABS-KEY ( "hospital day*" ) ) |  | 905,371 |
|  | Final Scopus Search: |  |  |  |
|  |  |  | ( TITLE-ABS-KEY ( "melatonin" ) )  AND  ( ( TITLE-ABS-KEY ( "intensive care unit*" ) )  OR  ( TITLE-ABS-KEY ( "intensive therap*" ) )  OR  ( TITLE-ABS-KEY ( "icu" ) )  OR  ( TITLE-ABS-KEY ( "itu" ) )  OR  ( TITLE-ABS-KEY ( "critical care unit*" ) ) )  AND  ( ( TITLE-ABS-KEY ( "length of stay" ) )  OR  ( TITLE-ABS-KEY ( "los" ) )  OR  ( TITLE-ABS-KEY ( "hospitali*ation" ) )  OR  ( TITLE-ABS-KEY ( "hospital stay" ) )  OR  ( TITLE-ABS-KEY ( "hospital day*" ) ) )  unit*" ) )  OR  ( TITLE-ABS-KEY ( "intensive therap*" ) )  OR  ( TITLE-ABS-KEY ( icu ) )  OR  ( TITLE-ABS-KEY ( itu ) ) ) | 199 |
