# Supplementary material for: The effect of exogenous melatonin and melatonin receptor agonists on intensive care unit and hospital length of stay: A systematic review and meta-analysis
Source: PLoS One. 2025 Sep 8;20(9):e0332031. doi: 10.1371/journal.pone.0332031 (PMC12416736; doi:10.1371/journal.pone.0332031)
Supplement: Table S4 — (DOCX) [file pone.0332031.s004.docx]

**Supplementary Table S4.** Data extraction table

| **Author(s), year of publication & country** | **Aim** | **Design** | **Setting** | **Sample (size, population, age)** | **Instrument** | **Relevant outcomes measures** | **Primary or secondary** | **Medication name, dose, and route of administration** | **Results** |
| --- | --- | --- | --- | --- | --- | --- | --- | --- | --- |
| 1. Abassi et al. (2018), Iran | “…to evaluate the effect of exogenous melatonin on delirium development and its related adverse sequelae in the subgroup of medical and surgical ICU patients.” pp - 1571. | Single centre, double-blinded, randomised, placebo-controlled trial | ICU in a university teaching hospital | Sample size n = 137  Melatonin group n = 67  Placebo group n = 70  Patients admitted to ICU >18 years old for more than 5 days and screened for delirium.  Age (years), mean ± SD:  Melatonin: 52.5 ± 18.4  Placebo: 49.9 ± 19  p = 0.46 | Not clear | ICU LOS  Hospital LOS | Secondary | Melatonin 3mg orally for 5 days. | ICU LOS (days) mean ± SD:  Melatonin group 8.8 ± 5.9  Placebo group 9.8 ± 10.6  p = 0.5  Hospital LOS (days), mean ± SD:  Melatonin group 18.1 ± 13.5  Placebo group 18.6 ± 15.6  p = 0.85 |
| 2. Ameri et al. (2023), Iran | “…evaluated the efficacy and safety of addition oral melatonin to standard treatment of hospitalized patients with COVID-19-induced pneumonia.” pp - 266 | single-center, open-label, randomized clinical trial with a parallel-group design | ICU in a university affiliated hospital | Final sample size n = 226  Melatonin group n = 109  Placebo group n = 117  Patients >20 years old admitted to ICU with confirmed COVID-19  Age (years), mean ± SD:  Melatonin: 54.6 ± 11.51  Control: 54.69 ± 13.4 | Not clear | ICU LOS  Hospital LOS | Secondary | Melatonin 5mg BD orally x 7 days | ICU LOS (days) median (IQR):  Melatonin: 9 (6-10)  Control: 13 (7-15)  p = <0.001  Hospital LOS:  Melatonin: 15 (13 – 17)  Control: 21 (14 – 24)  p = 0.026 |
| 3. Bandyopadhyay et al. (2024), India | “…to determine whether enteral melatonin decreases the incidence of delirium in critically ill adults.” pp - 2 | Single centre, open-label parallel group randomized controlled trial | mixed medical-surgical ICU of a tertiary care teaching hospital | Total sample size n = 108  Melatonin group n = 54  Control group n = 54  All patients 18- 65 with an expected ICU stay of > 24 hours.  Age (years) mean ± SD:  Melatonin: 34.7 ± 14.5  Control: 35.1 ± 14.2 | Jamovi version 1.6 based on R Core version 4.0. | ICU LOS | Secondary | 3mg of melatonin enterally at 9 PM for 7 consecutive days | ICU LOS (days) median (IQR):  Melatonin: 5 (4-9)  Control: 7.5 (5-11.75)  p = 0.1 |
| 4. Bellapart et al. (2020), Australia | To determine if melatonin supplementation “positively influences the sleep architecture and the incidence of delirium in  intensive care.” pp - 1. | Single centre,  double-blinded, randomised, placebo-controlled trial | ICU in a large teaching hospital | Final sample size n = 33  Melatonin group n = 21  Placebo group n = 12  Patients in the “recovery phase” of their disease, defined as “the resolution of the acute pathological process for which the patient had been admitted to ICU and the initiation of weaning from mechanical ventilation.” pp - 2.  Age (years), median (IQR):  Melatonin: 55 (43-66)  Placebo: 57.5 (50-64 | Metavision^TM^ Clinical Information System | ICU LOS | Secondary | 3mg melatonin capsules with water via NGT at 9pm followed by 0.5mg every hour until 3am (total 6mg/night) for 3 nights. | ICU LOS (days), median (IQR):  Melatonin group: 24 (16-36)  Placebo: 23 (16-35)  p = 0.71 |
| 4. Dianatkhah et al. (2017), Iran* | “… to evaluate the effect of  exogenous melatonin as an adjuvant on the duration of mechanical ventilation, length of ICU stay, and mortality  in intubated patients with hemorrhagic stroke.” pp – 173 & 174. | Single centre,  double-blinded, randomised, control trial | ICU in a university hospital | Final sample n = 40  Melatonin group n = 20  Control group n = 20  Adult patients admitted to ICU with acute, spontaneous intra-cerebral haemorrhage.  Age (years), mean ± SD:  Melatonin group: 57.7 ± 12.7  Control 52.9 ± 13.7  p = 0.329 | Not clear | ICU LOS | Primary | Melatonin 30mg nocte via NGT. | ICU LOS (days), median (IQR):  Melatonin: 8 (6-21)  Control:  12 (8-25)  p = 0.041 |
| 6. Gandolfi et al. (2020), Brazil | “To evaluate whether the use of exogenous melatonin  affects sleep, reduces the prevalence of delirium, and decreases  the need for analgosedation and to assess whether serum melatonin  indices correlate with exogenous administration in critically  ill patients.” pp – 1286. | Multi-centre, double-blinded, randomised, placebo-controlled trial | ICUs in two tertiary level hospitals | Final sample size n = 203  Melatonin group n = 102  Placebo group n = 101  Adult patients (≥18 years old) admitted to either of the participating ICUs for at least 1 night.  Age (years), mean ± SD:  Melatonin: 60 ± 14.8  Placebo: 57 ± 15.4  p = 0.344 | Hospital electronic medical record system | ICU LOS  Hospital LOS | Secondary | Melatonin 10mg orally or via NGT at 8pm (2 hours after dinner) for 7 days. | ICU LOS (days), median (IQR):  Melatonin: 4 (2-10)  Placebo: 4 (2-10)  p = 0.597  Hospital LOS (days), median (IQR):  Melatonin: 9 (4-17)  Placebo: 10 (5-22)  p = 0.221 |
| 7. Hakiminia et al. (2021), Iran | “…to evaluate the effects of melatonin, a mitochondria-targeted antioxidant, on mitochondrial and brain injury markers, and the clinical outcomes of patients with ABI [acquired brain injury].” pp – 118. | Single centre, double-blinded, randomised, placebo-controlled trial | ICU in a university hospital | Final sample size n = 60  Melatonin group n = 30  Placebo group n = 30  Patients ≥18 years old, diagnosed with TBI on non-TBI, identified within 72 hours of brain injury onset in the ICU or neurology ward.  Age (years), mean ± SD (range):  Melatonin: 46.1 ± 23.92 (18-97)  Placebo: 49.27 ± 19.96 (18-93)  p = 0.58 | Not clear | ICU LOS  Hospital LOS | Secondary | 21mg of melatonin (seven 3mg tablets) daily divided into 12mg in the morning and 9mg in the evening for 5 consecutive days. | ICU LOS (days), median (IQR):  Melatonin: 13 (9-22)  Placebo: 14 (6.75-27.25)  p = 0.987  Hospital LOS (days), median (IQR):  Melatonin: 16 (14-30)  Placebo: 19 (10.75-33.25)  p = 0.719 |
| 8. Jaiswal et al et al. (2019), USA | “To assess the efficacy of ramelteon in preventing delirium, an acute neuropsychiatric condition associated with increased morbidity and mortality, in the peri-operative, Intensive Care Unit (ICU) setting” pp – 1751. | Single centre, parallel-arm  double-blinded, randomised, placebo-controlled trial | ICU in an academic medical centre | Final sample size n = 117  Ramelteon group n = 59  Placebo group n = 58  Patients ≥18 years old admitted for elective pulmonary thromboendarterectomy.  Age (years), mean ± SD:  Ramelteon: 58.1 ± 14.1  Placebo: 56.1 ± 15.8  p = 0.471 | Not clear | ICU LOS  Hospital LOS | Secondary | Ramelteon 8mg orally or via NGT, once daily at 9pm beginning the night before surgery for a maximum of 7 nights while still in the ICU. | ICU LOS (days), median (IQR):  Ramelteon: 4 (3-6)  Placebo: 4 (3-5)  p = 0.349  Hospital LOS (days), median (IQR):  Ramelteon: 12 (10-16)  Placebo: 12 (10-14)  p = 0.72 |
| 9. Mahrose et al. (2021), Egypt | “…to assess the effect of supplementing dexmedetomidine infusion with oral melatonin in prevention of postoperative delirium after coronary artery bypass graft surgery.” pp – 62. | Single centre, randomised control trial | ICU at a university hospital | Final sample size n = 110  Melatonin group n = 55  Control group n = 55  Patients >60 years old undergoing elective CABG surgery.  Age (years), mean ± SD:  Melatonin: 67 ± 6.7  Control: 66.1 ± 6.3 | Not clear | ICU LOS  Hospital LOS | Secondary | 5mg melatonin tablets at 10pm the night before surgery and then every 24 hours for 3 days. | ICU LOS (days), mean ± SD:  Melatonin: 3.1 ± 1.4  Control: 3.6 ± 2.0  p = 0.16  Hospital LOS (days), mean ± SD:  Melatonin: 11.9 ± 5.3  Control 13.8 ± 6.2  p = 0.096 |
| 10. Mansilla-Rosello et al. (2022), Spain | “To determine whether IV melatonin therapy improves redox status and  inflammatory responses in surgical patients with severe sepsis” pp – 1. | Single centre,  double-blinded, randomised, placebo-controlled trial | ICU in a university hospital | Final sample size n = 29  Melatonin group n = 15  Placebo group n = 14  Adults (≥18 years old) with severe sepsis due to a disease requiring surgical intervention and admitted to the ICU.  Age (years), mean ± SEM:  Melatonin: 65.5 (43-88)  Placebo: 71.6 (51-87) | Not clear | Hospital LOS | Secondary | IV melatonin 60mg over 24 hours from the time of sepsis diagnosis following surgery and for 5 subsequent days. | Hospital LOS (days), mean:  Melatonin: 21.42  Placebo: 26.64 |
| 11. Mistraletti et al. (2015), Italy | “…to determine whether nocturnal melatonin supplementation would reduce the need for sedation in patients with critical illness.” pp - 1298 | Single centre, double-blinded, randomised, placebo-controlled trial | Mixed medical-surgical ICU of a university hospital | Final sample size n = 82  Melatonin group n = 41  Placebo group n = 41  Adult patients (≥18 years old) admitted to the general ICU with an expected LOS > 2 days and a predicted mortality at ICU admission of >13% (based on the Simplified Acute Physiology Score – SAPSII)  Age (years), mean ± SD:  Melatonin: 68 ± 15  Placebo: 65 ± 15  p = 0.28 | Not clear | ICU LOS | Secondary | 3mg melatonin at 8pm and midnight (from 3^rd^ day of admission to ICU) via NG/NJ tube or via ileostomy after crushing tablet and mixing in 20mL of water, followed by another 20mL water flush. | ICU LOS (days), median (IQR):  Melatonin: 14 (18-20)  Placebo: 12 (9-29)  p = 0.75 |
| 12. Nasseh et al. (2022), Iran | “…to evaluate melatonin's effect on cardiac biomarkers after coronary artery bypass grafting  (CABG).” pp – 3800. | Single centre, double-blinded, randomised, placebo-controlled trial | Cardiac surgery ICU in a university hospital | Final sample size n = 100  Melatonin group n = 50  Placebo group n = 50  Patients aged 50-80 years old who underwent elective CABG surgery.  Age (years), mean ± SD:  Melatonin: 62.8 ± 9.2  Placebo: 60.2 ± 8.6  p = 0.145 | Not Clear | ICU LOS  Hospital LOS | Secondary | 3mg the night before surgery, 3mg the morning of surgery, 3mg every night post-surgery for 3 nights. | ICU LOS (days), mean ± SD:  Melatonin: 3.4 ± 1.05  Placebo: 3.96 ± 1.06  p = 0.01  Hospital LOS (days), mean ± SD:  Melatonin: 7.94 ± 2.4  Placebo: 8.37 ± 3.2  p = 0.454 |
| 13. Nickkholgh et al. (2011), Germany | “To assess the safety of a preoperative single dose of  melatonin in patients undergoing major liver resection” pp – 381. | Single centre, double-blinded, randomised, placebo-controlled trial | ICU at a university hospital and transplant centre | Final sample size n = 36  Melatonin group n = 18  Placebo group n = 18  Patients aged 18 to 90 years old undergoing elective major partial liver resection.  Age (years), mean ± SEM:  Melatonin: 59 ± 10  Placebo: 56 ± 11  p = 0.4 | Not clear | ICU LOS  Hospital LOS | Secondary | Melatonin 50mg/kg of body weight in 250ml of milk via NGT preoperatively once the patient had been intubated. | ICU LOS (days), mean ± SEM:  Melatonin: 2.3 ± 1.5  Placebo: 3 ± 2.2  p = 0.2  Hospital LOS (days), mean ± SEM:  Melatonin: 13.5 ± 1.5  Placebo: 17 ± 2  p = 0.3 |
| 14. Nishikimi et al. (2018), Japan | “To examine whether the use of ramelteon,  a melatonin agonist, can prevent delirium and shorten the duration  of ICU stay of critically ill patients.” pp -1099. | Single centre, triple-blinded, randomised, placebo-controlled trial | Emergency and medical ICU (EMICU) of an academic hospital | Final ample size n= 88  Ramelteon group n = 45  Placebo group n = 43  Patients ≥ 20 years old admitted to the EMICU and commenced on treatment within 48 hours.  Age (years), median (IQR):  Ramelteon 68 (57-75)  Placebo 68 (52-78) | Not clear | ICU LOS | Primary | Ramelteon 8mg/day at 8pm each night until discharged from ICU | ICU LOS (days), median (IQR):  Ramelteon: 4.56 (2.1-7.07)  Placebo: 5.86 (2.97-14.16)  ICU LOS (log days), mean (SD):  Ramelteon: 1.5 (1.02)  Placebo: 1.86 (0.92)  p = 0.082 |
| 15. Sharifnia et al. (2021), Iran* | “to evaluate the neuroprotective effect of melatonin on patients with hemorrhagic stroke” pp 1 | Single centre,  double-blinded, randomised, control trial | ICU in a university hospital | Final sample n = 40  Melatonin group n = 20  Control group n = 20  Adult patients admitted to ICU with acute, spontaneous intra-cerebral haemorrhage.  Age (years), mean ± SD:  Melatonin group: 57.7 ± 12.7  Control 52.9 ± 13.7  p = 0.329 | Not clear | ICU LOS | Primary | Melatonin 30mg nocte via NGT. | ICU LOS (days), median (IQR):  Melatonin: 8 (6-21)  Control:  12 (8-25)  p = 0.041 |
| 16. Shi Y. (2021), China | “The present study explored whether acute Mel[atonin] treatment could reduce the incidence of delirium.” pp 893 | Double-blinded, randomised, parallel-arm, placebo-controlled trial. | ICUs in Jiangsu – unclear how many sites | Sample size n = 297  Melatonin group n = 148  Placebo group n = 149  Patients aged ≥60 years old who underwent PCI under GA and were admitted to ICU.  Age (years), mean ± SD  Melatonin: 71.5 ±6.7  Placebo: 71.6 ±6.6  p = 0.9 | Not clear | Hospital LOS* (results section describes hospital LOS but only ICU LOS is mentioned in the outcomes assessment | secondary | 3mg/day for 7 days post PCI | *results document ‘hospital stay” – table also reverses the results reported in the text of the results section.  (days), mean ± SD:  Melatonin 13.4 ± 6.6  Placebo 15.9 ± 9.7  p = 0.01 |
| 17. Soltani et al. (2022), Iran | “…to assess the effect of exogenous melatonin on the reduction in the need for sedative agents and the duration of mechanical ventilation in TICH patients in ICU.” | Single-centre, double-blind, randomized controlled study | Single ICU at a teaching hospital in Ahvaz, Iran | Sample size n = 52  Melatonin group n = 26  Control group n = 26  Patients aged ≥ 18 years with traumatic intracranial  haemorrhage confirmed by computer tomography  requiring surgery, initial GCS 4–8,  and expected length longer than 2 days.  Age (years), mean ± SD  Melatonin: 34.62 ± 16.23  Placebo: 36.85 ± 17.47 | SPSS software package 22.0 | ICU LOS | secondary | 3mg at 9pm during ICU stay | ICU LOS (days) mean ± SD:  Melatonin 13.31 ± 5.27  Control 14.55 ± 4.72  p = 0.482 |
| 18. Tirkan et al. (2024), Iran | “…to determine the effectiveness  of melatonin compared with placebo in critically ill patients  with COVID-19 who were admitted to the ICU.” | Single centre, double-blind randomised control trial | Single ICU at a university teaching hospital | Sample size n = 86  Melatonin group n = 44  Control group n = 42  Patients aged ˃20 years with  definite diagnosis of COVID-19, ability to sign a consent form and sufficient literacy, no use of melatonin during the study, no other systemic diseases, history of no allergy to melatonin, no use of anticoagulant drugs.  Age (years), mean ± SD  Melatonin: 58.02 ± 13.42  Placebo: 60.95 ± 13.18 | Microsoft Excel | ICU LOS  Hospital LOS | Secondary | Melatonin 10mg for 7 days | ICU LOS (days) mean ± SD:  Melatonin 11.23 ± 4.73  Placebo 11.9 ± 6.52  p = 0.582  Hospital LOS mean ± SD:  Melatonin 19.7 ± 8.77  Control 21.48 ± 10.85  p = 0.407 |
| 19. Vijayakumar et al. (2016), India | “to know the effect of melatonin on duration  of delirium and recovery profile in OPCP [organophosphate compound poisoning) patients.” pp 32 | Single-centre, double-blinded, randomised, placebo-controlled trial | Single ICU at a teaching hospital | Sample size n = 56  Melatonin group n = 26  Placebo group n = 30  Patients aged 18-50 years old admitted to ICU with organophosphate compound poisoning.  Age (years), mean ± SD  Melatonin: 38 ± 14.4  Placebo: 36.9 ± 10.3 | Not clear | ICU LOS | secondary | 3mg melatonin at 9pm daily for duration on ICU stay. | ICU LOS (days), mean ± SD:  Melatonin 7.65 ± 3.58  Placebo 9.36 ± 6.35  p = 0.21 |
| 20. Wibrow et al. (2022), Australia | “to determine whether administration of melatonin decreases the  prevalence of delirium in critically ill patients.” pp 414 | Multi-centre, double-blinded, randomised, placebo-controlled trial | 12 ICUs across Australia | Randomised n= 847  Melatonin group n = 419  Placebo group n = 422  Patients ≥18 years old who required ICU admission with an expected LOS > 72 hours.  Age (years), mean ± SD:  Melatonin: 61.9 ± 15.1  Placebo: 61.9 ±15.2 | Not clear | ICU LOS  Hospital LOS | secondary | 4mg melatonin (oral or via NGT) at 9pm for 14 consecutive nights or until discharge from ICU, whichever occurred first. | ICU LOS (days), median (IQR):  Melatonin 5 (4-8)  Placebo 5(3-7)  p = 0.135  Hospital LOS, median (IQR):  Melatonin 14 (9-21)  Placebo 12 (8-20)  p = 0.816 |
| 21. Zadeh et al. (2021), Iran | “…”to evaluate  the effect of melatonin administration on the  inhibition of delirium following on-pump coronary artery bypass graft (CABG)” | Single centre, double-blind, randomised control trial | ICU in a University affiliated hospital | Sample size n = 60  Melatonin group n = 30  Control group n = 30  Patients >30 years old who were candidates for on-pump coronary artery bypass grafting, with a minimum ejection fraction of 30%  Age (years), mean ± SD  Melatonin: 60.26 ± 9.5  Placebo: 62.9 ± 8.08 | SPSS software version 22.0 | ICU LOS | Secondary | 3mg | ICU LOS (days) mean ± SD:  Melatonin: 3.87 ± 1  Control: 4 ± 1.7  p = 0.04 |

*Indicates the two separate papers published on the same patient cohort by Dianatkhah et al. and Sharifnia et al. (2021)

SD = standard deviation; IQR = interquartile range; ICU = Intensive care unit; LOS = length of stay; NGT = nasogastric tube; GA = general anaesthetic
