# Supplementary material for: The effect of exogenous melatonin and melatonin receptor agonists on intensive care unit and hospital length of stay: A systematic review and meta-analysis
Source: PLoS One. 2025 Sep 8;20(9):e0332031. doi: 10.1371/journal.pone.0332031 (PMC12416736; doi:10.1371/journal.pone.0332031)
Supplement: Table S5 — (DOCX) [file pone.0332031.s005.docx]

**Supplementary Table S5.** Breakdown of meta-analysis results by disease group with the two statistical outliers.

|  | **ICU length of stay (LOS)** | | | | | | **Hospital length of stay (LOS)** | | | | | | | |
| --- | --- | --- | --- | --- | --- | --- | --- | --- | --- | --- | --- | --- | --- | --- |
| **By disease group** | **Descriptive** | | **Effect size (LOS difference)** | | **Heterogeneity** | | **Descriptive** | | | **Effect size (LOS difference)** | | | **Heterogeneity** | |
| Subgroups | **studies** | **patients** | **mean (95% CI)** | ***p*-value** | **I^2^ (95% CI)** | ***p*-value** | **Studies** | **patients** | **mean (95% CI)** | | ***p*-value** | **I^2^ (95% CI)** | | ***p*-value** |
| **General ICU** | 7 | 1492 | -0.89 (-2.25, 0.46) | 0.197 | **69% (31%, 86%)** | **0.004** | 3 | 1181 | -0.17 [ -2.72; 2.37] | | **0.895** | 60% (0%, 88%) | | 0.084 |
| Outlier 1: Wibrow et al., 2022 | 1 | 841 | **0.70 (0.30, 1.10)** | **< 0.001** | - | - | 1 | 841 | **1.30 (0.09, 2.51)** | | **0.035** | - | | - |
| Other studies | 6 | 651 | **-1.44 (-2.74, -0.15)** | **0.029** | 8% (0%, 77%) | 0.367 | 2 | 340 | -1.85 (-4.49, 0.78) | | 0.169 | 0% | | 0.519 |
| **Coronary artery bypass graft surgery** | 3 | 270 | **-0.47 (-0.78; -0.16)** | **0.003** | 0% (0%, 90%) | 0.643 | 2 | 210 | -0.86 (-2.18, 0.45) | | 0.198 | 29% | | 0.235 |
| **COVID-19** | 2 | 312 | -2.20 (-4.74, 0.34) | 0.090 | 72% (0%, 94%) | 0.057 | 2 | 312 | **-3.90 (-6.28, -1.51)** | | **0.001** | 35% | | 0.214 |
| Tirkan et al. 2024 | 1 | 86 | -0.67 (-3.09, 1.75) | 0.587 | - | - | 1 | 86 | -1.78 (-5.96, 2.40) | | 0.404 | - | | - |
| Outlier 2: Ameri et al. 2023 (COVID-19) | 1 | 226 | **-3.30 (-4.52, -2.07)** | **< 0.001** | - | - | 1 | 226 | **-4.59 (-6.06, -3.12)** | | **<0.001** | - | | - |
| **Other disease-specific** | 6 | 361 | -0.48 (-1.76, 0.80) | 0.466 | 0% (0%, 75%) | 0.519 | 5 | 539 | -1.83 (-4.16, 0.50) | | 0.125 | **66% (12%, 87%)** | | **0.019** |
| Elective Pulmonary Thromboendarterectomy | 1 | 117 | 0.35 (-0.35, 1.05) | 0.322 | - | - | 1 | 117 | 0.71 (-0.69, 2.11) | | 0.322 | - | | - |
| Acquired Brain Injuries | 1 | 60 | -1.36 (-8.08, 5.37) | 0.693 | - | - | 1 | 60 | -0.86 (-8.51, 6.79) | | 0.825 | - | | - |
| Major liver resection | 1 | 36 | -0.70 (-5.92, 4.52) | 0.793 | - | - | 1 | 36 | -3.50 (-8.40, 1.40) | | 0.161 | - | | - |
| Haemorrhagic stroke | 1 | 40 | -3.28 (-11.17, 4.61) | 0.415 | - | - | - | - | - | | - | - | | - |
| Organophosphorus compound poisoning | 1 | 56 | -1.71 (-4.37, 0.95) | 0.207 | - | - | - | - | - | | - | - | | - |
| Traumatic intracranial hemorrhage | 1 | 52 | -1.24 (-3.93, 1.45) | 0.366 | - | - | - | - | - | | - | - | | - |
| Percutaneous transluminal coronary intervention | - | - | - | - | - | - | 1 | 297 | -2.50 (-4.39, -0.61) | | 0.009 | - | | - |
| Surgical patients with severe sepsis | - | - | - | - | - | - | 1 | 29 | -5.22 (-10.15, -0.29) | | 0.038 | - | | - |
| **All studies** | **18** | **2435** | **-0.83 (-1.52, -0.15)** | **0.017** | **73% (57%, 83%)** | **< 0.001** | **12** | **2242** | **-1.56 (-2.88, -0.24)** | | **0.020** | **79% (63%, 88%)** | | **<0.001** |

**Abbreviations:** ICU = Intensive care unit; LOS = length of stay.
