# Supplementary material for: The effect of exogenous melatonin and melatonin receptor agonists on intensive care unit and hospital length of stay: A systematic review and meta-analysis
Source: PLoS One. 2025 Sep 8;20(9):e0332031. doi: 10.1371/journal.pone.0332031 (PMC12416736; doi:10.1371/journal.pone.0332031)
Supplement: Table S6 — (DOCX) [file pone.0332031.s006.docx]

**Supplementary Table S6.** Breakdown of meta-analysis results by medication type with the two statistical outliers.

|  | **ICU length of stay (LOS)** | | | | | | **Hospital length of stay (LOS)** | | | | | | | |
| --- | --- | --- | --- | --- | --- | --- | --- | --- | --- | --- | --- | --- | --- | --- |
| **By medication type** | **Descriptive** | | **Effect size (LOS difference)** | | **Heterogeneity** | | **Descriptive** | | | **Effect size (LOS difference)** | | | **Heterogeneity** | |
| Subgroups | **studies** | **patients** | **mean (95% CI)** | ***p*-value** | **I^2^ (95% CI)** | ***p*-value** | **Studies** | **patients** | **mean (95% CI)** | | ***p*-value** | **I^2^ (95% CI)** | | ***p*-value** |
| **Melatonin studies** | 16 | 2230 | -0.86 (-1.58, -0.13) | 0.020 | 73% | <0.001 | 11 | 2125 | -1.86 (-3.24, -0.49) | | 0.008 | 78% | | <0.001 |
| Without statistical outliers | 14 | 1163 | -0.55 (-0.84, -0.25) | < 0.001 | 0% | 0.903 | 9 | 1058 | -1.71 (-2.78, -0.63) | | 0.002 | 0.5% | | 0.429 |
| Outlier 1: Wibrow et al., 2022 (General ICU) | 1 | 841 | 0.70 (0.30, 1.10) | < 0.001 | - | - | 1 | 841 | 1.30 (0.09, 2.51) | | 0.035 | - | | - |
| Outlier 2: Ameri et al. 2023 (COVID-19) | 1 | 226 | -3.30 (-4.52, -2.07) | < 0.001 | - | - | 1 | 226 | -4.59 (-6.06, -3.12) | | <0.001 | - | | - |
| **Ramelteon studies** | 2 | 205 | -1.16 (-4.60, 2.29) | 0.510 | 83% | 0.015 | 1 | 117 | 0.71 [-0.69; 2.11] | | 0.322 | - | | - |
| Elective Pulmonary Thromboendarterectomy | 1 | 117 | 0.35 (-0.35, 1.05) | 0.322 | - | - | 1 | 117 | 0.71 [-0.69; 2.11] | | 0.322 | - | | - |
| General ICU | 1 | 88 | -3.20 (-5.99, -0.41) | 0.025 | - | - | - | - | - | | - | - | | - |
| **All studies** | **18** | **2435** | **-0.83 (-1.52, -0.15)** | **0.017** | **73%** | **< 0.001** | **12** | **2242** | **-1.56 (-2.88, -0.24)** | | **0.020** | **79%** | | **<0.001** |
| **Total without the two statistical outliers** | **16** | **1368** | **-0.49 (-0.88, -0.10)** | **0.015** | **7%** | **0.370** | **10** | **1175** | **-1.34 (-2.47, -0.22)** | | **0.019** | **38%** | | **0.107** |

**Abbreviations:** ICU = Intensive care unit; LOS = length of stay.
