# Supplementary material for: The effect of exogenous melatonin and melatonin receptor agonists on intensive care unit and hospital length of stay: A systematic review and meta-analysis
Source: PLoS One. 2025 Sep 8;20(9):e0332031. doi: 10.1371/journal.pone.0332031 (PMC12416736; doi:10.1371/journal.pone.0332031)
Supplement: Table S7 — (DOCX) [file pone.0332031.s007.docx]

**Supplementary Table S7.** Bias-corrected meta-results results.

|  | **ICU length of stay (LOS)** | | | | | **Hospital length of stay (LOS)** | | | | | | |
| --- | --- | --- | --- | --- | --- | --- | --- | --- | --- | --- | --- | --- |
| **Result from data** | **Descriptive** | | **Effect size (LOS difference)** | | **Heterogeneity** | **Descriptive** | | | **Effect size (LOS difference)** | | | **Heterogeneity** |
| Bias-adjusted estimates | **studies** | **patients** | **mean (95% CI)** | ***p*-value** | **I^2^ (95% CI) or**  ***p*-values*** | **Studies** | **patients** | **mean (95% CI)** | | ***p*-value** | **I^2^ (95% CI) or**  ***p*-values*** | |
| All studies | **18** | **2435** | **-0.83 (-1.52, -0.15)** | **0.017** | **73% (57%, 83%)** | **12** | **2242** | **-1.56 (-2.88, -0.24)** | | **0.020** | **79% (63%, 88%)** | |
| Trim and fill estimate | 26 (+8) | 3147 | -0.08 (-0.87, 0.70) | 0.832 | 77% (67%, 84%) | 18 (+6) | 3143 | 0.23 (-1.30, 1.75) | | 0.773 | 88% (82%, 92%) | |
| Limit meta-analysis estimate | 18 | 2435 | -0.59 (-1.39, 0.21) | 0.147 | Small *p* < 0.001  Other *p* < 0.001 | 12 | 2242 | -1.08 (-2.83, 0.67) | | 0.225 | Small *p* = 0.012  Other *p* < 0.001 | |
| Melatonin (no outliers) | **16** | **1368** | **-0.49 (-0.88, -0.10)** | **0.015** | **7% (0%, 43%)** | **10** | **1175** | **-1.34 (-2.47, -0.22)** | | **0.019** | **38% (0%, 70%)** | |
| Trim and fill estimate | 22 (+6) | 1802 | -0.31 (-0.71, 0.09) | 0.131 | 22% (0%, 54%) | 15 (+5) | 1850 | -0.16 (-1.37, 1.05) | | 0.796 | 62% (33%, 78%) | |
| Limit meta-analysis estimate | 16 | 1368 | -0.25 (-0.72, 0.22) | 0.298 | Small *p* < 0.077  Other *p* < 0.523 | 10 | 1175 | -0.61 (-2.20; 0.99) | | 0.456 | Small *p* = 0.028  Other *p* = 0.292 | |

**Abbreviations:** ICU = Intensive care unit; LOS = length of stay.

*The “small *p*” is a *p*-value for a test of small-study effects. The “Other *p*” is a p-value for test of residual heterogeneity beyond small-study effects.
